# Outdoor cultivation of *Picochlorum* sp. in a novel V-shaped photobioreactor on the Caribbean island Bonaire

**DOI:** 10.3389/fbioe.2024.1347291

**Published:** 2024-06-13

**Authors:** Rocca Chin-On, Mila de Boer, Cas van de Voort, Juliëtte Camstra, Maria Barbosa, René H. Wijffels, Marcel Janssen

**Affiliations:** ^1^ Bioprocess Engineering and AlgaePARC, Wageningen University and Research, Wageningen, Netherlands; ^2^ Water-en Energiebedrijf Bonaire, Kralendijk, Netherlands; ^3^ Biosciences and Aquaculture, Nord University, Bodø, Norway

**Keywords:** microalgae, photobioreactor, outdoor cultivation, biomass productivity, photoinhibition, temperature

## Abstract

Microalgae are a promising renewable feedstock that can be produced on non-arable land using seawater. Their biomass contains proteins, lipids, carbohydrates, and pigments, and can be used for various biobased products, such as food, feed, biochemicals, and biofuels. For such applications, the production costs need to be reduced, for example, by improving biomass productivity in photobioreactors. In this study, *Picochlorum* sp. (*BPE23*) was cultivated in a prototype of a novel outdoor V-shaped photobioreactor on Bonaire (12°N, 68°W). The novel photobioreactor design was previously proposed for the capture and dilution of sunlight at low-latitude locations. During several months, the biomass productivity of the local thermotolerant microalgae was determined at different dilution rates in continuous dilution and batch dilution experiments, without any form of temperature control. Reactor temperatures increased to 35°C–45°C at midday. In the continuous dilution experiments, high average biomass productivities of 28–31 g m^−2^ d^−1^ and photosynthetic efficiencies of 3.5%–4.3% were achieved. In the batch dilution experiments, biomass productivities were lower (17–23 g m^−2^ d^−1^), as microalgal cells likely experienced sudden light and temperature stress after daily reactor dilution. Nonetheless, dense cultures were characterized by high maximum photosynthetic rates, illustrating the potential of *Picochlorum* sp. for fast growth under outdoor conditions.

## 1 Introduction

Microalgae are photosynthetic microorganisms that typically grow in aquatic environments, such as brackish and seawater. The biomass of microalgae contains proteins, lipids, carbohydrates, and pigments, and can potentially be used as a renewable feedstock for various biobased products, including food, feed, chemicals, and biofuels (Chisti, 2008; Becker, 2013; Borowitzka, 2013; Khan et al., 2018; Williamson et al., 2024). In the last decades, the cultivation of microalgae for such purposes has been studied and developed at different scales (Zittelli et al., 2013; Rios Pinto et al., 2021). A major advantage is that microalgae cultivation does not require arable land or freshwater and that cultivation conditions can be regulated for optimal growth. Nevertheless, the production costs of microalgae still need to be reduced further to enable the commercialization of microalgal products, particularly bulk products (Acién et al., 2012; Ruiz et al., 2016). One approach to reducing production costs is to improve biomass productivity in photobioreactors.

Photobioreactors are closed cultivation systems in which microalgae can grow by means of photosynthesis. Cultivation conditions such as biomass concentration, pH, CO_2_ supply, and nutrient availability can be adjusted and regulated. When suitable conditions and sufficient nutrients are provided, the predominant factor that determines biomass productivity in photobioreactors is light; i.e., algal growth is typically light-limited (Richmond, 2013; Janssen, 2016). Therefore, geographical locations that are characterized by an abundance of sunlight throughout the year are favorable for microalgae cultivation (Ugwu et al., 2008). Moreover, light capture by a photobioreactor itself must be maximized through appropriate reactor design. Losses of light from cultivation systems because of reflection or absorption by the ground must be limited, for instance by optimizing the orientation of photobioreactor units as well as the distance between them (Qiang et al., 1998; Slegers et al., 2011).

In addition to light availability, the intensity of light that the microalgal culture in a photobioreactor receives is of importance as it determines the efficiency with which light is converted into chemical energy in biomass (i.e., the photosynthetic efficiency). Most microalgae are adapted to low light intensities between 50 and 300 μmol PAR photons m^−2^ s^−1^ (Janssen, 2016), while the intensity of sunlight can reach almost 2,000 μmol PAR photons m^−2^ s^−1^ around solar noon. When exposed to such high light intensities, the photosystems of microalgae become oversaturated. In that case, not all photons that are absorbed can be utilized, and the photosynthetic efficiency decreases as excess photons are dissipated (i.e., photo-saturation) (Janssen, 2016). Photoinhibition may even occur depending on the microalgae and the light intensity, leading to inactivation or damage of the photosystems and a reduction in photosynthesis (Tredici, 2010; Janssen, 2016). Theoretically, the photosynthetic efficiency has been estimated to be between 6% and 10% for microalgae (Tredici, 2010; Janssen, 2016), though much lower long-term efficiencies, below 3%, have been observed in practice (De Vree et al., 2015). To improve photosynthetic efficiencies, the photo-saturation effect needs to be reduced and photoinhibition needs to be prevented by diluting sunlight to lower intensities in outdoor photobioreactors. To achieve light dilution, studies have been carried out on lenses or mirrors in combination with optical fibers or light guides for redistribution of light within the culture (Janssen et al., 2003; Zijffers et al., 2008; Zittelli et al., 2013), as well as on reactor geometries that enable strong refraction of sunlight at midday (Tredici and Zlttelli, 1998; Cuaresma et al., 2011; Zittelli et al., 2013; De Vree et al., 2015; Cho et al., 2019).

In an attempt to improve the efficiency with which sunlight is utilized for biomass production, we investigated the cultivation of microalgae in a novel outdoor V-shaped PBR on Bonaire (Chin-On et al., 2022). Bonaire (12°N, 68°W) is an island in the Caribbean that is characterized by high irradiance and a stable climate throughout the year, which are favorable conditions for microalgae cultivation. Recently, a novel V-shaped photobioreactor design was proposed for microalgae cultivation at low-latitude locations to simultaneously capture and dilute available sunlight, thereby reducing losses of light from the system and exposing the microalgae culture to lower and more favorable light intensities (Chin-On et al., 2022). Based on model simulations, it was found that significant gains of up to 40% in biomass productivity and photosynthetic efficiency could be achieved in V-shaped photobioreactors compared to a horizontal photobioreactor, primarily as a result of light dilution around noon (Chin-On et al., 2022). Sunlight was found to be highly diluted during the day due to refraction at the surfaces of the reactor panels, particularly in panels with large inclination angles (Chin-On et al., 2022).

Another important factor that influences the growth of microalgae is temperature. Most microalgae grow optimally at a temperature between 20°C and 30°C. However, temperatures in outdoor photobioreactors can fluctuate and become much higher during the day due to the absorption of sunlight (Wang et al., 2012; Ras et al., 2013). When optimal temperatures of microalgae are exceeded, growth rates are known to decline rapidly because of heat stress on cells, which affects photosynthetic processes and can be lethal (Ras et al., 2013). To prevent overheating, photobioreactors need to be cooled, for example, by shading parts of the reactor, spraying reactor surfaces with fresh water (i.e., evaporative cooling), or using heat exchangers (Wang et al., 2012; Pruvost et al., 2016). Nevertheless, shading can limit light availability and thereby affect biomass productivity, whereas spraying the reactor and using heat exchangers can be expensive in terms of water or energy use (Pruvost et al., 2016; Huang et al., 2017). Costs for temperature control in photobioreactors were found to contribute significantly to the overall production costs of microalgae and need to be reduced, for instance, through passive forms of cooling, the use of reflective materials, and strain improvement (Pruvost et al., 2016).

In an attempt to minimize temperature control costs, we cultivated and analyzed the growth of thermo-tolerant *Picochlorum* sp. *BPE23* in our outdoor photobioreactor, without any form of active cooling. In previous research, the green microalgal strain *Picochlorum* sp. was isolated from samples of saltwater bodies on Bonaire and selected based on high growth rate in strain enrichment experiments at a temperature of 40°C during daytime and 30°C during night time (Barten et al., 2020). The strain showed optimal growth at a temperature of 40°C while being able to withstand peak temperatures up to 47.5°C under fluctuating diel temperature regimes (Barten et al., 2021). *Picochlorum* species have received increasing interest in research in recent years, due to their relatively high growth rates, their ability to survive under dynamic cultivation conditions, and their ability to tolerate high light intensities, temperatures, and salinity levels (Weissman et al., 2018; Gonzalez-Esquer et al., 2019; Barten et al., 2021; Krishnan et al., 2021). As a result of its thermo-tolerance, the cultivation of *Picochlorum* sp. may enable a reduction in production costs of more than 25% (Barten et al., 2021). Moreover, from analyses of the biomass composition of *Picochlorum* sp., a high protein content of 58% as well as a high level of fatty acid unsaturation, namely, 64% polyunsaturated fatty acids, was found, indicating the potential suitability of this microalga for food purposes (Barten et al., 2020).

The goal of this study was to determine the biomass productivity of thermotolerant *Picochlorum* sp. that can be achieved in practice in a novel V-shaped photobioreactor on Bonaire, to analyze the growth of this local microalga in the reactor under outdoor conditions, and to evaluate the overall performance of the system. To do so, a prototype with a V-shaped photobioreactor design was built and tested on Bonaire. With this prototype, we performed continuous dilution and batch dilution experiments using *Picochlorum* sp. over the period of several months, during which multiple dilution rates were tested while the biomass productivity was determined. Furthermore, to gain insight into the growth of the microalga, photosynthetic parameters including the maximum photosynthetic rate and the dark-adapted quantum yield of PSII photochemistry were analyzed during the batch dilution experiments.

## 2 Materials and methods

To determine the biomass productivity of *Picochlorum* sp. that can be achieved in a novel V-shaped photobioreactor on Bonaire (12°N, 68°W), continuous dilution and batch dilution experiments were performed with an outdoor prototype over periods of several months at AlgaePARC Bonaire. During the batch dilution experiments, several photosynthetic parameters were analyzed to gain insight into the growth of the microalga under outdoor conditions. The island Bonaire is located slightly north of the equator and receives sunlight from the south during most of the year.

### 2.1 Photobioreactor design and setup

**FIGURE 1:**
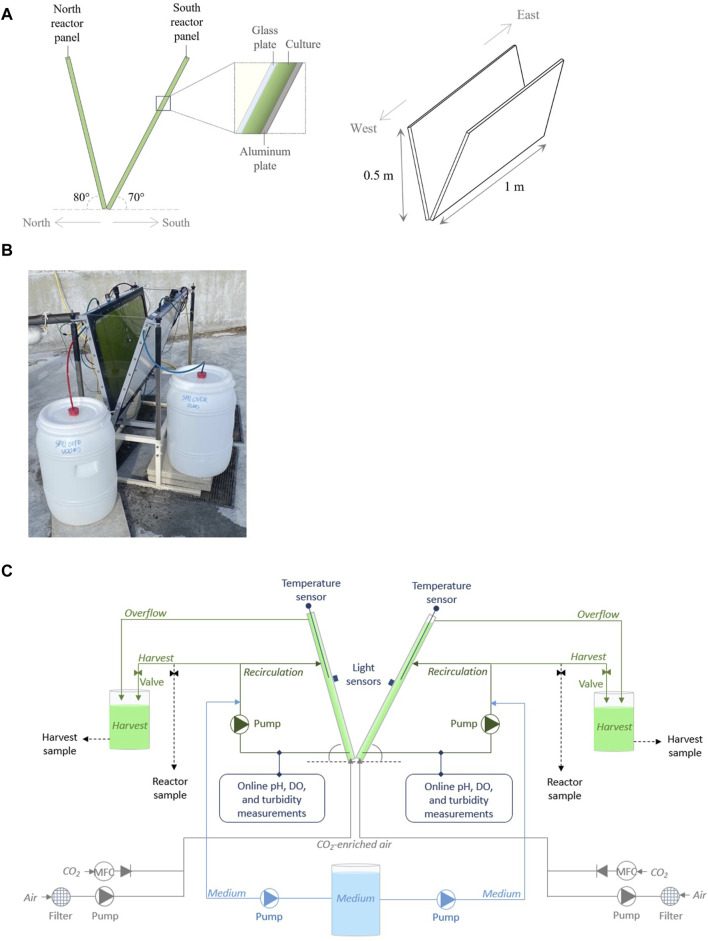
The design of the V-shaped photobioreactor **(A)** and a photograph of the prototype at AlgaePARC Bonaire **(B)**. Moreover, a schematic overview of the prototype, illustrating the north and south reactor panels as separate cultivation systems **(C)**.

The novel V-shaped photobioreactor that we investigated was introduced in a previous study (Chin-On et al., 2022) and consists of two inclined reactor panels that are arranged in a V-shaped configuration (Figure 1). One reactor panel is positioned to point in the north direction, its illuminated surface facing south, while the other panel is positioned to point in the south direction, its illuminated surface facing north (Figure 1A).

For this study, a prototype of a V-shaped photobioreactor was constructed (Figure 1B). The prototype was approximately 1 m long, 0.3 m wide, and 0.5 m high, and was held in place by a metal frame (Figure 1B). The reactor panel pointing north (hereafter referred to as “north reactor panel”) was inclined at an angle of 80°, and the reactor panel pointing south (hereafter referred to as “south reactor panel”) was inclined at an angle of 70° (Figure 1A). In model simulations, this V-shaped design was found to be favorable for microalgae cultivation on Bonaire in terms of areal biomass productivity and photosynthetic efficiency compared to V-shaped designs with smaller reactor panel inclination angles (Chin-On et al., 2022). Both reactor panels consisted of a glass plate, where the culture was exposed to sunlight, and an aluminum back plate (Figures 1A,B). The distance between these plates (i.e., the culture thickness) was 0.016 m. The technical drawing and dimensions of the prototype are presented in Supplementary Material S1.

The north and south reactor panels formed separate systems of 10 and 11 L, respectively, in which microalgae were cultivated (Figures 1B,C). The panels were monitored and operated individually as the microalgal cultures were exposed to different light conditions, leading to different growth rates. Each reactor panel was equipped with a sparger at the bottom of the panel, through which air (3 L min^−1^) and CO_2_ were supplied to the cultures (Figure 1C). Furthermore, each reactor panel was connected to an external recirculation loop, which functioned as a bypass, through which a small volume of the culture (2.8 L) was continuously pumped (Figure 1C). In these external loops, the cultures were supplied with an artificial seawater-based growth medium (Section 2.2). In addition, sensors were placed in the recirculation loops to continuously monitor and log pH (Polilyte Plus PHI Arc 120, Hamilton) and dissolved oxygen (VisiFerm DO Arc 120 H2, Hamilton) in the cultures. To regulate the pH within the cultures at a value of 7.0, CO_2_ was automatically supplied to the reactor panels based on the pH measurements. Moreover, a temperature sensor (Pt100, Endress + Hauser) was placed in the center of each reactor panel to measure the temperature within the culture, and a light sensor (Li-Cor SA-190 2*π* quantum sensor) was placed in the center on each of the glass plates to measure the irradiance on the panels (Figure 1C). A temperature and a light sensor were also placed near the prototype to measure the ambient temperature and irradiance on a horizontal surface. Lastly, the microalgae cultures could be harvested from the reactor panels via a valve in each recirculation loop or an overflow tube near the top of each panel (Figure 1C).

### 2.2 Microalgae and medium composition

In this research, we cultivated and analyzed the growth of green microalga *Picochlorum* sp. *(BPE23)* in our outdoor prototype. The microalga was isolated in a previous study (Barten et al., 2020)and grown in artificial seawater enriched with 20 ml L^−1^ nitrogen and phosphorus nutrient solution, 1 ml L^−1^ trace mineral solution, and 1 ml L^−1^ iron solution. The artificial seawater consisted of 29.2 g L^−1^ NaCl, 3.2 g L^−1^ Na_2_SO_4_, 0.85 g L^−1^ K_2_SO_4_, 0.075 g L^−1^ MgCl_2_·6H_2_O, and 0.0028 g L^−1^ CaCl_2_·2H_2_O. The nitrogen and phosphorus nutrient solution contained 37.5 g L^−1^ urea, 5.8 g L^−1^ KH_2_PO_4_, 7.4 g L^−1^ K_2_HPO_4_, and 3.0 g L^−1^ Na_2_EDTA, and its pH was adapted to 7.5. The trace element solution contained 45 g L^−1^ Na_2_EDTA·2H_2_O, 1.7 g L^−1^ MnCl_2_·2H_2_O, 0.66 g L^−1^ ZnSO_4_·7H_2_O, 0.07 g L^−1^ Co(NO_3_)_2_·6H_2_O, 0.024 g L^−1^ CuSO_4_·5H_2_O, 0.24 g L^−1^ Na_2_MoO_4_·2H_2_O, 0.013 g L^−1^ H_2_SeO_3_, 0.026 g L^−1^ NiSO_4_·6H_2_O, 0.018 g L^−1^ Na_3_VO_4_, and 0.019 g L^−1^ K_2_CrO_4_, and its pH was adapted to 5. The iron solution contained 39.6 g L^−1^ NaFeEDTA.

To start cultivating microalgae in the prototype, a pre-culture of *Picochlorum* sp. *(BPE23)* was used as inoculum. The inoculum was prepared in the lab in 2 L bottles, in which *Picochlorum* sp. was cultivated in the described medium, adapted to a pH of 7.0, at 28°C. 0.66 g L^−1^ NaHCO_3_ was added to the cultures. The bottles were illuminated by artificial light at an intensity of ∼30 μmol m^−2^ s^−1^ (PAR, 400–700 nm) for 12 h per day to mimic a 12/12 h day/night cycle. The bottles were also supplied with 400 ml min^−1^ of ambient air and 8 ml min^−1^ of CO_2_ (2% v/v).

The medium for the experiments in the prototype was prepared in batches of 60 L. The pH in the reactor panels of the prototype was controlled by the automatic addition of CO_2_ to the continuous airflow, which was used for mixing the cultures and stripping the oxygen. We aimed for a time-averaged 2% v/v CO_2_ concentration. 0.66 g L^−1^ NaHCO_3_ was added to the medium as this is the bicarbonate concentration in equilibrium with a gas phase of 2% v/v CO_2_ at pH 7.0. Once prepared, the medium was sparged with CO_2_ to prevent an increase in pH and precipitation of salts. During this period, a volume of CO_2_ of at least 5 times the headspace of the medium vessel was supplied to the medium. The medium was maintained under a CO_2_ headspace before use in the reactor panels.

### 2.3 Reactor operation and experiments

#### 2.3.1 Continuous dilution

In the continuous dilution experiments, three experimental runs of approximately 2 weeks were conducted, with a 1-week adaptation period in between each run. In each run, a different dilution rate was tested in the reactor panels (Table 1). Per run, the dilution rates in the north and south panels were nearly identical (Table 1).

**TABLE 1:** The experimental runs and the corresponding dilution rates that were applied in the reactor panels.

Operation mode	Run	Dilution rates in north and south reactor panel [% of reactor panel volume harvested per day]	Duration of run [days]	Month and year
Continuous dilution	1	33 and 31	18	November–December 2020
	2	18 and 15	11	December 2020
	3	54 and 52	10	January 2021
Batch dilution	1	60 and 53	21	October–November 2021
	2	44 and 40	19	November 2021
	3	34 and 27	15	December 2021
	4	20 and 15	17	January–February 2022

At the beginning of every run, the reactor panels were filled with medium, to which a pre-culture of *Picochlorum* sp. was introduced. Then, for several days, the reactor was operated in batch mode. Once the biomass concentration had increased and the cultures were a dark shade of green, the reactor panels were operated in continuous dilution mode for 1.5–2.5 weeks. The dilution rates were regulated by adjusting the pump speeds, which determined the flow of fresh medium into the reactor panels during the daytime hours. When the fresh medium was introduced to the reactor panels, the cultures were simultaneously harvested in separate vessels via the overflow tubes near the top of the panels.

Every morning at 9.30 a.m. (GMT-4), samples were taken from the reactor panels and the harvest vessels. In addition, the harvest of each panel was weighed to accurately assess the reactor dilution.

#### 2.3.2 Batch dilution

In the batch dilution experiments, four experimental runs of 2–3 weeks were conducted, with a 1-week adaptation period in between each run. In each run, a different daily reactor dilution was tested in the reactor panels (Table 1). The daily reactor dilutions of the north and south panels were not identical (Table 1).

To start the first run, the reactor panels were filled with medium, to which a pre-culture of *Picochlorum* sp. was added. Then, for several days, the reactor was operated in batch mode. Once the biomass concentration had increased and the cultures were a dark shade of green, the reactor panels were operated in batch dilution mode for 2–3 weeks. In this operation mode, a pre-determined volume of the cultures in the reactor panels was harvested every day at 3.00 p.m. (GMT-4), to achieve the desired reactor dilution. The cultures were harvested in separate vessels via the recirculation loops and the panels were subsequently filled with fresh medium. The first week of every run was used to stabilize the biomass concentration in the reactor panels after harvesting and diluting.

The same microalgal cultures were continuously used in the reactor panels for the first three runs. For the fourth run, the reactor panels were cleaned, filled with fresh medium, and inoculated with a new pre-culture of *Picochlorum* sp.

Every day after harvesting and diluting the reactor panels at 3.00 p.m. (GMT-4), samples were taken from the harvested cultures and the reactor panels, and the harvest of each panel was weighed.

### 2.4 Determining biomass concentration and productivity

The daily samples taken during the continuous dilution and batch dilution experiments were used to determine the biomass concentration of the cultures in the reactor panels and harvest vessels, based on optical density (OD) measurements and dry weight analyses.

#### 2.4.1 OD and dry weight measurements

The optical density of all samples was measured in duplicate at a wavelength of 720 nm using a fluorometer (AquaPen-C 100, Photon System Instruments). The optical density at this wavelength (OD_720_) quantifies light absorption related to light scattering of cells and is a measure of cell density.

Furthermore, the dry weight concentrations of samples were determined in triplicate using glass microfiber filters (Whatman, diameter 55 mm, pore size 0.7 μm). Clean filters were first dried overnight in an oven at 95°C, after which they were placed in a desiccator for at least 30 min and subsequently weighed. The filters were then used to filter samples containing about 3 mg biomass (dry weight) under mild vacuum conditions with 0.5 M ammonium formate (Zhu and Lee, 1997). Ammonium formate was used to rinse away salts from the biomass (Zhu and Lee, 1997). Samples were first diluted with ∼25 ml ammonium formate and the filters were pre-washed with ammonium formate before filtration. Ammonium formate was also used to rinse the glass and wash the biomass on the filter three times at the end of filtration. Afterward, filters were again dried overnight in an oven at 95°C, placed in a desiccator for at least 30 min, and weighed. The dry weight biomass concentration of a sample was calculated by subtracting the weight of the clean filter from the weight of the filter after filtration of the sample and dividing this difference by the volume of the sample.

During the continuous dilution experiments, dry weight concentrations were determined from filter analyses for samples taken on Mondays, Wednesdays, and Fridays. During the batch dilution experiments, filter analyses were performed for samples taken on all days, except Saturdays. For samples taken on the other days, the dry weight concentrations were derived from the correlation found between OD_720_ values and the dry weight concentrations on the previous and subsequent days. Average values and standard deviations were calculated. Standard deviations are presented as error bars in the results.

#### 2.4.2 Calculations

From the determined biomass concentrations of the cultures in the reactor panels and harvest vessels and the weight of the harvest, the biomass productivity in each reactor panel on a given day was calculated (Eq. 1):

$$r_{x,panel}^{t=1}=C_{x,panel}^{t=1}∙V_{panel}+C_{x,harvest}^{t=1}∙V_{harvest}^{t=1}-C_{x,panel}^{t=0}∙V_{panel}$$ (1)

In which $r_{x,panel}^{t=1}$ is the biomass productivity in a reactor panel on a given day (g d^−1^), $C_{x,panel}^{t=1}$ is the biomass concentration in the reactor panel (g L^−1^), $V_{panel}$ is the volume of the reactor panel (L), $C_{harvest}^{t=1}$ is the biomass concentration of the harvest (g L^−1^), $V_{harvest}^{t=1}$ is the volume of the harvest (L), and $C_{x,panel}^{t=0}$ is the biomass concentration in the reactor panel on the previous day (g L^−1^). The volume of the harvest, $V_{harvest}^{t=1}$ , was calculated based on its weight, assuming a density equal to that of water. The harvested biomass (g) was calculated as the product of the harvest volume $V_{harvest}^{t=1}$ (L) and the biomass concentration of the harvest $C_{harvest}^{t=1}$ (g L^−1^).

Furthermore, the biomass productivity of the entire V-shaped prototype on a given day (g·d^−1^) was calculated as the sum of the biomass productivities in both reactor panels (Eq. 2). The areal and volumetric biomass productivity of the prototype, in g m^−2^ d^−1^ and g L^−1^ d^−1^, were calculated by dividing the productivity of the prototype by its occupied horizontal surface area and its total volume (Supplementary Material S1), respectively (Eqs 3, 4).

$$r_{x,PBR}=r_{x,northpanel}^{t=1}+r_{x,southpanel}^{t=1}$$ (2)

$$r_{x,areal}=\frac{r_{x,PBR}}{A_{PBR}}$$ (3)

$$r_{x,vol}=\frac{r_{x,PBR}}{V_{PBR}}$$ (4)

In which $r_{x,PBR}$ (g d^−1^) is the biomass productivity of the photobioreactor on a given day, $r_{x,northpanel}^{t=1}$ and $r_{x,southpanel}^{t=1}$ (g d^−1^) are the biomass productivities in the north and south reactor panel, $r_{x,areal}$ (g m^−2^ d^−1^) is the areal biomass productivity of the photobioreactor, $A_{PBR}$ (0.33 m^2^) is the horizontal surface area of the photobioreactor, $r_{x,vol}$ (g L^−1^ d^−1^) is the volumetric biomass productivity of the photobioreactor, and $V_{PBR}$ (21.5 L) is the volume of the cultures in the photobioreactor.

Lastly, the biomass yield on light (g_x_ mol_ph_^−1^) was calculated by dividing the areal biomass productivity of the prototype on a given day by the total PAR irradiance that was incident on a horizontal surface during that day (mol_ph_ m^−2^ d^−1^) (Eq. 5). The latter was measured with a light sensor on a horizontal surface near the reactor.

$$Y_{x/ph}=\frac{r_{x,areal}}{I_{ph,hor}}$$ (5)

In which $Y_{x/ph}$ (g_x_ mol_ph_^−1^) is the biomass yield on light, $r_{x,areal}$ (g m^−2^ d^−1^) is the areal biomass productivity of the photobioreactor, and $I_{ph,hor}$ (mol_ph_ m^−2^ d^−1^) is the irradiance measured on a horizontal surface.

The photosynthetic efficiency (Eq. 6) was determined from the areal biomass productivity by first calculating the energy content of the produced biomass on each day and the energy content of the measured irradiance on the same day. The following conversion factors were used: 4.6 µmol-PAR photons × J-PAR photons^−1^, 24 g mol-biomass^−1^, and 5.59 × 10^5^ J mol-biomass^−1^ (Janssen, 2016). The photosynthetic efficiency over the complete sunlight spectrum was then calculated as the fraction of the energy content of the produced biomass over the available light energy, assuming that the PAR fraction is equal to 42.5% of the complete sunlight spectrum.

$$PE=\frac{\frac{r_{x,areal}}{24}∙5.59∙10^{5}}{\frac{I_{ph,hor}∙10^{6}}{4.6}}∙0.425∙100$$ (6)

In which PE (%) is the photosynthetic efficiency, $r_{x,areal}$ (g m^−2^ d^−1^) is the areal biomass productivity of the photobioreactor, and $I_{ph,hor}$ (mol_ph_ m^−2^ d^−1^) is the irradiance measured on a horizontal surface.

### 2.5 Determining photosynthetic parameters

During the batch dilution experiments, the maximum photosynthetic rate and the dark-adapted quantum yield of PSII photochemistry of the *Picochlorum* sp. cultures in the reactor panels were determined (Henley, 1993) to gain insight into the growth of the microalga under outdoor conditions.

#### 2.5.1 Maximum photosynthetic rate

The maximum photosynthetic rate was determined from oxygen production measurements using a liquid-phase biological oxygen monitor (BOM) (Oxytherm+, Hansaetech). For these measurements, samples of the cultures in the reactor panels were taken at 10.00 a.m., 1.00 p.m., and 3.00 p.m. (GMT-4) on 8 days of every batch dilution run. The north and south panels were each sampled on four separate days. The short-term oxygen production in these samples was measured in duplicate. On different days, measurements were performed at different temperatures, namely, at 25°C, 32°C, and 39°C. Measurements at 39°C were performed on two separate days. As such, the short-term oxygen production (μmol_O2_ m^−3^ s^−1^) of the sampled cultures of both reactor panels was determined at different biomass concentrations, different times of the day, and different temperatures.

Before measurements, the electrode of the BOM was calibrated at 100% air saturation using medium equilibrated with air, followed by 0% O_2_ saturation through the addition of sodium dithionite. The sample in the cuvette was then prepared and contained the sampled culture from the reactor panels with additional medium including 4.77 g L^−1^ HEPES buffer at pH 7.0, and 50 μl 0.5 M NaHCO_3_. The total liquid volume in the cuvette was set at 2.4 ml. The ratio of medium and sample volume was chosen such that the biomass concentration in the cuvette was approximately 0.1 g_x_ L^−1^. The cuvette was closed with a conical stopper preventing any gaseous headspace, and the liquid was continuously stirred. The BOM measurement was performed in a sequence of increasing light intensity during a series of time intervals (Supplementary Material S2).

From the measurements, the specific oxygen production rate (μmol_O2_ g_x_^−1^ s^−1^) was calculated by dividing the oxygen production rate (μmol_O2_ L^−1^ s^−1^), measured by the BOM, by the biomass concentration of the sample (g_x_ L^−1^). The maximum specific oxygen production rate was then determined by plotting the specific oxygen production against light intensity in photosynthesis-irradiance (PI) curves. The initial slopes of these curves were also analyzed.

#### 2.5.2 Dark-adapted quantum yield of PSII photochemistry

The dark-adapted quantum yield (QY) of PSII photochemistry of the cultures in the reactor panels was measured using a fluorometer (AquaPen-C 100, Photon System Instruments). For these measurements, samples were taken from the reactor panels at 9.30 a.m., 1.00 p.m., and 3.00 p.m. on 2 days of every batch dilution run. The north and south panels were sampled on the same days. On each of the 2 days, measurements were performed at different temperatures, namely, at 25°C and 39°C. As such, the dark-adapted QY of PSII photochemistry of the sampled cultures of both reactor panels was determined during the different runs, at different times of the day, and different temperatures.

Before measurements, samples were stored in the dark for 15 min, after which the culture was transferred to a cuvette and placed in the fluorometer. In the device, the minimum fluorescence was first measured. Then the samples were exposed to a short, intense, and saturating light pulse during which the maximum fluorescence was measured. From these measurements, dark-adapted QY was automatically calculated.

## 3 Results

### 3.1 Continuous dilution experiments

#### 3.1.1 Environmental conditions

During the three continuous dilution runs, the daily irradiance on a horizontal surface amounted to 31–35 mol PAR photons m^−2^ d^−1^ on average (Table 2). Nevertheless, during each of the experimental runs, differences of more than 10 mol PAR photons m^−2^ d^−1^ were found between the days, illustrating the variability of irradiance within the same period. In general, irradiance reached its peak between 10.30 a.m. and 2.00 p.m., at which peak light intensities of 1,800–2,300 μmol PAR photons m^2^ s^−1^ were measured (Supplementary Material S3).

**TABLE 2:** Measured irradiance in mol PAR photons m^−2^ d^−1^ on a horizontal surface, the north reactor panel, and the south reactor panel (top) during the continuous dilution experiments. Measured peak light intensities in μmol PAR photons m^−2^ s^−1^ during the day. Measured ambient and reactor panel temperatures in °C (bottom). Due to technical errors in data logging, irradiance and temperature data were incomplete on days 8, 9, 11, 14, 17, and 18 of run 1, days 5, 6, and 11 of run 2, and day 3 of run 3. Data on these days were not incorporated in the results shown below.

Irradiance	Horizontal		North reactor panel		South reactor panel	
[mol PAR photons m^−2^ d^−1^]	Average	Range	Average	Range	Average	Range
Run 1	30.7	19.8–38.3	10.9	5.0–16.6	4.5	3.5–5.4
Run 2	35.2	27.8–40.7	15.2	9.5–19.8	4.4	4.0–5.0
Run 3	34.3	26.4–42.4	15.8	11.4–23.7	4.8	4.2–5.0

Peak light intensity	Horizontal		North reactor panel		South reactor panel	
[μmol PAR photons m^−2^ s^−1^]	Average	Range	Average	Range	Average	Range
Run 1	2,117	1,986–2,393	1,096	994–1,252	292	251–364
Run 2	2,169	2,049–2,286	1,319	1,229–1,376	287	206–355
Run 3	2090	1,805–2,321	1,349	1,329–1,436	283	227–320

Temperature	Ambient		North reactor panel		South reactor panel	
[°C]	Average	Range	Average	Range	Average	Range
Run 1	27.5	22.5–32.6	27.0	21.2–38.3	28.2	22.9–36.0
Run 2	27.6	22.0–32.5	28.2	20.1–41.0	28.9	21.5–37.9
Run 3	26.3	22.4–31.5	26.6	20.5–39.2	27.1	21.6–35.1

Moreover, the irradiance measured on the reactor panels gives an indication of the incident sunlight on the panels but is not an exact quantification as spatial differences can occur over the panels as a result of shading and reflection of light. The north reactor panel was found to receive more sunlight compared to the south panel (Table 2). This was caused by the orientation of the north reactor panel, which was facing south. Since the experiments were performed in winter months, during which the sun has a more southern position relative to the Earth, the north reactor panel was exposed to more irradiance than the south reactor panel. Peak light intensities on the north reactor panel were 1,000–1,400 μmol PAR photons m^−2^ s^−1^, and peak light intensities on the south reactor panel were 200–350 μmol PAR photons m^−2^ s^−1^ (Supplementary Material S3). The measurements show that sunlight was indeed diluted by the V-shaped photobioreactor design as light intensities on the inclined surfaces were lower compared to those on a horizontal surface.

Furthermore, the ambient temperature was 26°C–28°C on average, though it fluctuated between 22°C and 33°C during the day (Table 2), indicating a variation of up to 11°C within 24 h. In general, peak ambient temperatures of 31°C–33°C were reached between 10.30 a.m. and 2.30 p.m., after which these temperatures declined (Supplementary Material S3). Ambient temperatures during the night between 7.00 p.m. and 7.00 a.m. were relatively stable at a value between 22°C and 28°C (Supplementary Material S3). After sunrise, ambient temperatures increased again. The ambient temperature and its progression correlated with irradiance; on days and hours with more irradiance, higher ambient temperatures were measured.

In the reactor panels, average temperatures were slightly higher (27°C–29°C) than the ambient temperature. Nevertheless, temperature fluctuations in the reactor panels were significantly larger, especially in the north reactor panel (Table 2). Variations of up to 15°C–20°C were measured during a single day. Temperatures in the north reactor panel increased up to 41°C, while temperatures in the south reactor panel increased up to 38°C (Table 2). In general, these peak reactor temperatures were reached between 11.00 a.m. and 3.00 p.m. (Supplementary Material S3). Both reactor panels heated up during the day, as the irradiance and the ambient temperature increased, and sunlight was absorbed by the cultures. At the end of the afternoon, reactor temperatures declined with irradiance (Supplementary Material S3), and heat was released from the panels. During the night between 7.00 p.m. and 7.00 a.m., temperatures were similar to, or slightly lower than, the ambient temperature (Supplementary Material S3). The average temperatures in the reactor panels were similar to each other, and the temperature ranges in each reactor panel were similar for all runs (Table 2).

#### 3.1.2 Reactor dilution and biomass productivity

**FIGURE 2:**
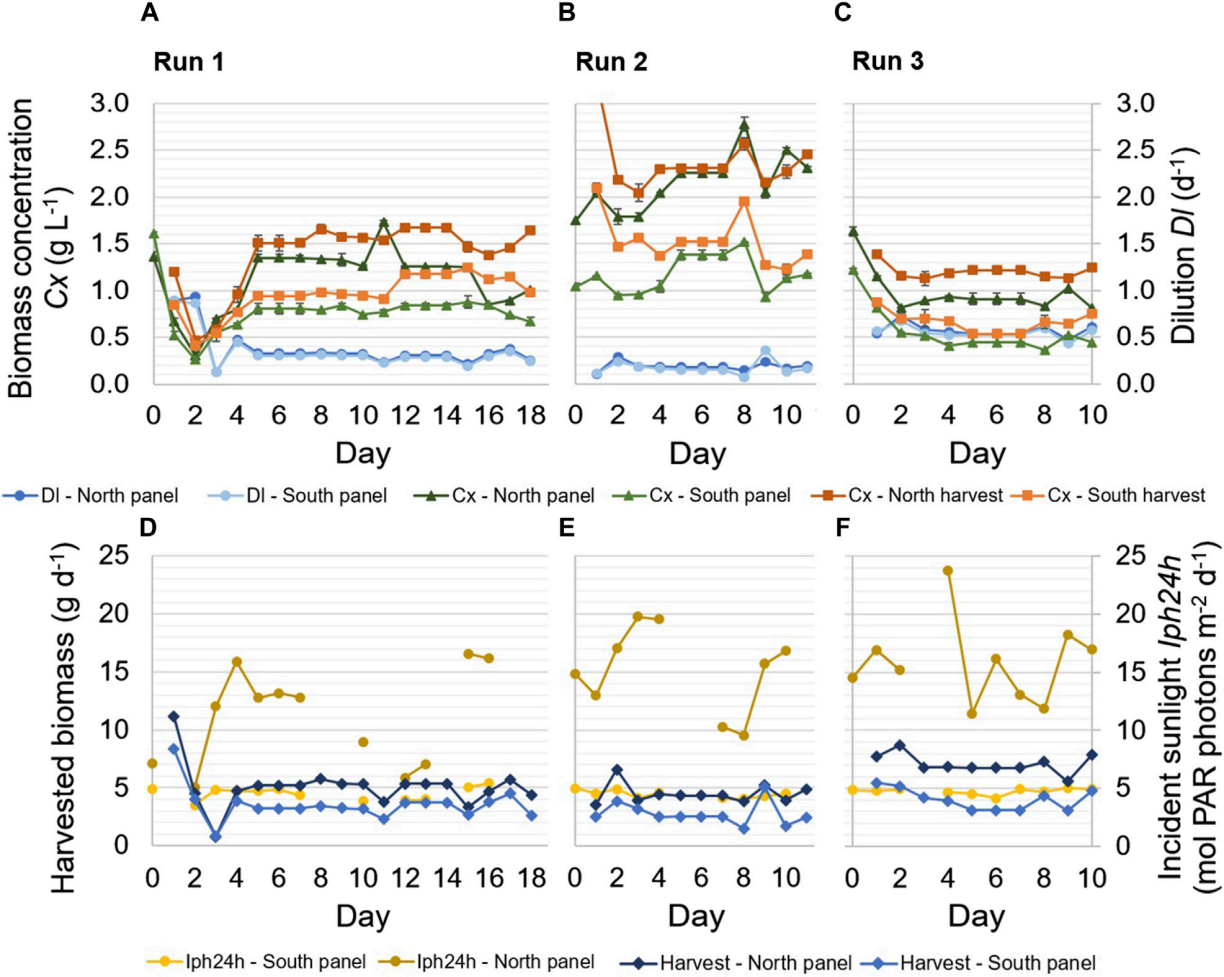
Experimental results of three continuous dilution runs. Samples of the reactor panels and the harvested cultures were taken every day at 9.30. Applied dilution rates Dl (d^−1^) in the reactor panels, as well as biomass concentrations C_x_ (g L^−1^) in the reactor panels and the harvest of the panels, are shown during the runs **(A–C)**. The error bars related to the biomass concentrations indicate the standard deviation of the measurements of a sample. In addition, the daily incident sunlight Iph24h (mol PAR photons m^−2^ d^−1^) on the reactor panels and the daily amount of harvested biomass (g d^−1^) are shown **(D–F)**.

In the continuous dilution experiments, different dilution rates were applied in the reactor panels in three runs of 1.5–2.5 weeks (Table 1; Figures 2A–C). During each of the runs, a pseudo-steady-state was achieved, in which the dilution rate was constant on several subsequent days (Figures 2A–C; Table 3). On the other days, dilution rates varied, primarily as a result of technical difficulties related to the pumps or clogging of the tubes.

**TABLE 3:** Overview of the average dilution rate (d^−1^), biomass productivity (g L^−1^ d^−1^ and g m^−2^ d^−1^), and photosynthetic efficiency (%) achieved during steady-state periods within the three continuous dilution runs. Average biomass productivities are presented per reactor panel (g L^−1^ d^−1^) and for the entire prototype (g m^−2^ d^−1^). The average photosynthetic efficiency was calculated from the steady-state days on which irradiance data was available.

	Pseudo-steady-state days	Average dilution rate			Average biomass productivity			
		North panel	South panel	North panel		South panel	Prototype–areal	Prototype–PE
		[d^−1^]	[d^−1^]	[g L^−1^ d^−1^]		[g L^−1^ d^−1^]	[g m^−2^ d^−1^]	[%]
Run 1	Day 5–10	0.33	0.31	0.59		0.31	29.1	4.3
Run 2	Day 4–7	0.18	0.15	0.54		0.33	28.3	3.5
Run 3	Day 4–7	0.54	0.52	0.66		0.30	30.8	4.2

The highest dilution rate in the reactor panels resulted in the lowest biomass concentration, and the lowest dilution rate resulted in the highest biomass concentration (Figures 2A–C). An increase in dilution rate resulted in a decrease in biomass concentration, as more biomass was harvested and more medium was supplied, and *vice versa* (Figure 2). The biomass concentration of the harvested culture from each reactor panel was determined from the harvest that was collected over a day. It therefore represents an average of the biomass concentration in the respective reactor panel during that 1 day. The biomass concentration in the reactor panels refers to the biomass concentration of the reactor cultures at the time of sampling (i.e., 9.30 a.m. every morning) and was lower compared to the concentration of the harvest (Figures 2A–C). While the dilution rates in the north and south reactor panels were nearly identical to each other in all runs, the biomass concentration in the north reactor panel was significantly higher than that in the south reactor panel. This difference in biomass concentration was caused by the larger amount of incident sunlight on the north reactor panel (Table 2; Figures 2D–F), which enabled a higher volumetric biomass productivity in that panel.

The amount of incident sunlight on the north reactor panel was also more variable during the runs compared to that on the south reactor panel (Figures 2D–F). Such fluctuations were also observed in the measured ambient irradiance on a horizontal surface near the prototype (Table 2), illustrating the variable weather conditions during the months of the experimental runs. The south reactor panel was exposed to less sunlight, likely of a more diffuse and consistent nature, as its illuminated surface faced north. On some days of the runs, data on incident sunlight was incomplete, due to interruptions in data logging (Figures 2D–F).

The amount of biomass that was harvested each day (Figures 2D–F) was the product of the dilution rate and biomass concentration in the reactor panel. A decrease in dilution rate, for instance on days 11 and 15 of run 1 and day 8 of run 2, typically resulted in a decrease in harvested biomass (Figure 2). On the other hand, an increase in dilution rate, for instance on day 9 of run 2 and day 9 of run 3, typically resulted in an increase in harvested biomass (Figure 2). In run 3, during which the highest dilution rates were applied, more biomass was harvested on most days compared to runs 1 and 2. Furthermore, an increase in dilution rate also corresponded to a decrease in biomass concentration, and *vice versa* (Figures 2A–C).

In Figures 2D–F, the harvested biomass from the reactor panels is shown instead of the biomass productivity (Eq. 1). The biomass productivity (Eq. 1) includes an additional term that accounts for the accumulation of biomass in the reactor panels and was found to be highly variable during a run. The variation was likely caused by errors in taking representative samples from the reactor panels, due to the release of biofilm within tubing into the samples. Such errors are avoided in the determination of the harvested biomass, and the harvested biomass therefore gives better insight into the long-term productivity in the reactor panels.

In Table 3, an overview of the average biomass productivity in the individual reactor panels and the complete prototype (i.e., north and south reactor panels combined) is given for the three continuous dilution runs. These average biomass productivities were determined for the pseudo-steady-state periods of each run, during which the dilution rate was constant over several subsequent days (Figures 2A–C; Table 3).

For the north reactor panel, the biomass productivity increased with the dilution rate (Table 3). The highest biomass productivity in this panel was achieved at a dilution rate of 0.54 days^−1^, at which the lowest biomass concentrations were measured (Figures 2A–C). At higher biomass concentrations, the denser culture may have been more photo-limited due to self-shading of microalgal cells, resulting in larger dark zones and lower biomass productivities. For the south reactor panel, the biomass productivity was similar among the runs and applied dilution rates. The highest biomass productivity was achieved at a dilution rate of 0.15 days^−1^, at which the highest biomass concentrations were measured (Figure 2). At higher dilution rates, more wash-out of biomass likely occurred, and the thinner culture may have been slightly more subjected to photo-inhibition. Nevertheless, it must be noted that for both reactor panels, the differences in average biomass productivity between the three runs were relatively small; up to 0.12 g L^−1^ d^−1^ in the north reactor panel and only 0.03 g L^−1^ d^−1^ in the south reactor panel (Table 3).

The average areal biomass productivity of the prototype was in a similar range among the three runs, namely, 28–31 g m^−2^ d^−1^ (Table 3). The highest areal biomass productivity in the complete prototype was found during run 3, corresponding to the highest dilution rates, in which an average areal biomass productivity of 30.8 g m^−2^ d^−1^ was achieved (Table 3). In this run, the average biomass yield on light was 0.92 g mol PAR photons^−1^ (Table 3), corresponding to an average photosynthetic efficiency of 4.2%.

### 3.2 Batch dilution experiments

#### 3.2.1 Environmental conditions

During the four batch dilution runs (Table 1), the daily irradiance on a horizontal surface amounted to 33–35 mol PAR photons m^−2^ d^−1^ on average (Table 4). Irradiance differences of 15–25 mol PAR photons m^−2^ d^−1^ were found during a single run, illustrating the variability of irradiance within the same period (Table 4). In general, irradiance reached its peak between 10.00 a.m. and 2.00 p.m., at which peak light intensities of 1,900–2,400 μmol PAR photons m^−2^ s^−1^ were measured on a horizontal surface.

**TABLE 4:** Measured irradiance in mol PAR photons m^−2^ d^−1^ on a horizontal surface, the north reactor panel, and the south reactor panel (top) during the batch dilution experiments. Measured peak light intensities in μmol PAR photons m^−2^ s^−1^ during the day. Measured ambient temperature in °C, and measured temperatures in the north and south reactor panels (bottom). Irradiance and/or temperature data was incomplete on days 9, 12, 14, and 22 of run 1, days 5 and 9–13 of run 3, and days 9 and 14 of run 4. Data on these days were not incorporated in the results shown below.

Irradiance	Horizontal		North reactor panel		South reactor panel	
[mol PAR photons m^−2^ d^−1^]	Average	Range	Average	Range	Average	Range
Run 1	35.0	16.6–42.2	13.4	4.3–20.6	8.1	4.1–10.7
Run 2	32.5	15.0–41.0	11.7	2.7–19.1	4.8	3.0–7.8
Run 3	32.8	23.1–38.3	11.7	6.3–14.6	4.9	3.8–5.8
Run 4	35.2	21.7–44.4	12.0	5.9–18.8	4.3	3.0–5.5

Peak light intensity	Horizontal		North reactor panel		South reactor panel	
[μmol PAR photons m^−2^ s^−1^]	Average	Range	Average	Range	Average	Range
Run 1	2,160	1,966–2,476	1,007	917–1,165	507	337–680
Run 2	2,038	1,866–2,286	1,032	850–1,153	399	285–518
Run 3	2,120	1,881–2,369	1,043	984–1,123	443	333–534
Run 4	2,253	2,017–2,397	1,056	979–1,165	321	266–408

Temperature: [°C]	Ambient		North reactor panel		South reactor panel	
	average	range	average	range	average	range
**Run 1**	28.5	23.6-34.8	32.4	25.7-45.1	29.7	23.4-39.8
**Run 2**	27.9	23.3-34.2	31.7	25.0-44.0	29.0	22.7-38.4
**Run 3**	27.5	23.5-32.5	30.8	25.3-42.3	28.2	23.5-36.5
**Run 4**	26.3	21.8-32.4	30.2	23.5-44.0	27.5	21.5-37.5

The north reactor panel received more sunlight compared to the south panel (Table 4), as its surface faced south during the period of the batch dilution experimental runs. Peak light intensities on the north reactor panel were 900–1,200 μmol PAR photons m^−2^ s^−1^, and peak light intensities on the south reactor panel were 300–550 μmol PAR photons m^−2^ s^−1^. Sunlight was diluted by the V-shaped photobioreactor design as the light intensities on the reactor panel surfaces were lower than the light intensities measured on a horizontal surface.

Moreover, the average ambient temperature was 26°C–29°C, and a variation of up to 11°C was found within a single day (Table 4). In general, peak ambient temperatures of 32°C–35°C were reached between 11.00 a.m. and 2.30 p.m., after which these temperatures declined. The ambient temperature gradually decreased during the night between 7.00 p.m. and 5.30 a.m. to a value between 22°C and 28°C. After sunrise, the ambient temperature increased again.

In the reactor panels, average temperatures were slightly higher (28°C–32°C) than the average ambient temperature (Table 4). Variations in reactor temperatures of up to 20°C were measured during a single day, and temperature fluctuations were larger in the north reactor panel than in the south reactor panel. Temperatures in the north reactor panel increased up to 45°C, while temperatures in the south reactor panel increased up to 40°C (Table 4). In general, peak reactor temperatures were reached between 11.00 a.m. and 3.00 p.m. as sunlight was absorbed by the cultures. At the end of the afternoon, reactor temperatures declined with the irradiance. During the night between 7.00 p.m. and 7.00 a.m., temperatures in the south reactor panel were similar to the ambient temperature, while temperatures in the north reactor panel were slightly higher. The temperature ranges in both reactor panels differed slightly among the four runs (Table 4).

#### 3.2.2 Reactor dilution and biomass productivity

**FIGURE 3:**
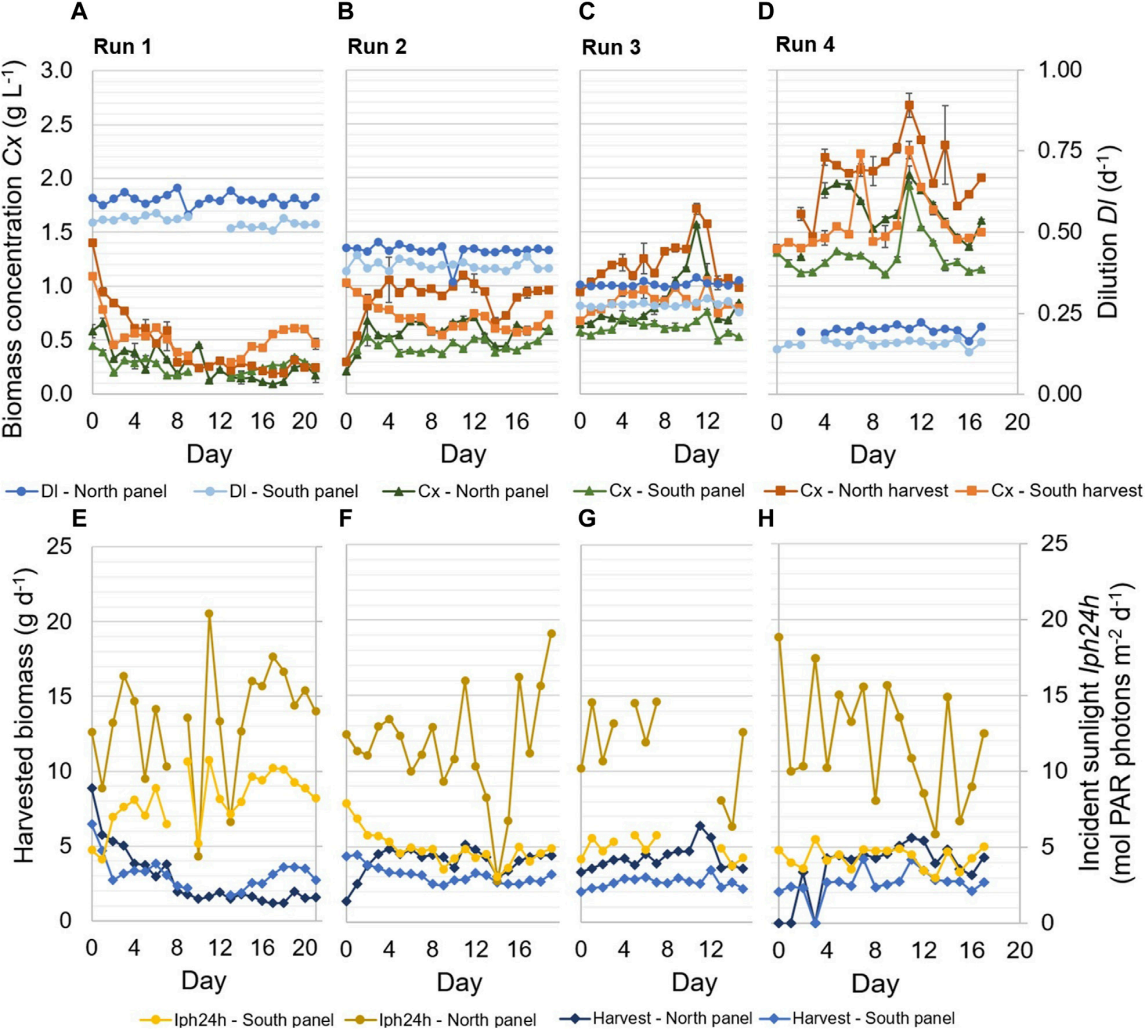
Experimental results of four repeated-batch runs. Samples of the reactor panels were taken after dilution at 15.00. Applied dilution rates Dl (d^−1^) in the reactor panels, as well as biomass concentrations C_x_ (g L^−1^) in the reactor panels and the harvest of the panels, are shown during the runs **(A–D)**. The error bars related to the biomass concentrations indicate the standard deviation of the measurements of a sample. In addition, the daily incident sunlight Iph24h (mol PAR photons m^−2^ d^−1^) on the reactor panels and the daily harvested biomass (g d^−1^) are shown **(E–H)**.

In the batch dilution experiments, different daily reactor dilutions were applied in the reactor panels in four experimental runs of 2–3 weeks (Table 1; Figures 3A–D). During each of the runs, a pseudo-steady-state was achieved, in which the dilution was constant on several subsequent days (Figures 3A–D; Table 5). On some days, reactor dilutions varied, cultures were not harvested, or data were missing (Figure 3), due to technical issues related to the reactor equipment, leakages, or data logging.

**TABLE 5:** Overview of the average dilution rate (d^−1^), biomass productivity (g L^−1^ d^−1^ and g m^−2^ d^−1^), and photosynthetic efficiency PE (%) achieved during the batch dilution runs. Average biomass productivities are given per reactor panel (g L^−1^ d^−1^) and for the entire prototype (g m^−2^ d^−1^). The average photosynthetic efficiency was calculated from the steady-state days on which irradiance data was available.

	Psuedo-steady-state days	Average reactor dilution			Average biomass productivity			
		North panel	South panel	North panel		South panel	Prototype–areal	Prototype–PE
		[d^−1^]	[d^−1^]	[g L^−1^ d^−1^]		[g L^−1^ d^−1^]	[g m^−2^ d^−1^]	[%]
Run 1	Day 0–21	0.60	0.53	0.25		0.26	16.7	2.0
Run 2	Day 0–19	0.44	0.40	0.41		0.30	23.0	3.2
Run 3	Day 0–15	0.34	0.27	0.43		0.23	21.3	2.6
Run 4	Day 5–17	0.20	0.16	0.40		0.31	23.4	3.1

The highest dilution of the reactor panels resulted in the lowest biomass concentrations, and biomass concentrations increased over the runs as decreasing reactor dilutions were applied (Figures 3A–D). The biomass concentrations of the harvested cultures were higher than the biomass concentrations of the cultures in the reactor panels since the latter were determined after dilution. During all runs, the daily dilution applied in the north reactor panel was slightly higher than the dilution applied in the south reactor panel (Figures 3A–D). Still, the biomass concentrations in the north reactor panel and its harvest were higher than those of the south reactor panel during most runs (Figures 3A–D), due to the larger amount of incident sunlight on the north reactor panel (Figures 3E–H).

The amount of incident sunlight measured on the north reactor panel was more variable during the runs compared to the incident sunlight measured on the south reactor panel (Figures 3E–H). The measurements reflect the variable weather conditions during the experimental runs (Table 4), which especially influenced the amount of irradiance received by the north reactor panel (Figures 3E–H). On some days, a correlation can be seen between the incident sunlight measured on the reactor panels and the amount of biomass harvested from those panels. For example, near the end of runs 1 and 2, the irradiance measured on the reactor panels increased for several days, which was followed by an increase in the biomass concentrations and the amount of biomass harvested (Figure 3).

Furthermore, the amount of biomass that was harvested each day was the product of the reactor dilution and biomass concentration in the reactor panel. A decrease in reactor dilution resulted in a decrease in harvested biomass, for instance on day 10 during run 2 (Figures 3B,F). A decrease in reactor dilution also corresponded to an increase in biomass concentration, and *vice versa* (Figures 3A–D). In Figures 3E–H, the harvested biomass from the reactor panels is shown instead of the biomass productivity (Eq. 1), as the harvested biomass gave more insight into the long-term productivity of the reactor panels.

Overall, the highest amount of biomass was harvested in run 4, during which the lowest daily reactor dilutions were applied (Figures 3D,H). Furthermore, the amount of biomass harvested from the north reactor panel was higher than that from the south reactor panel during most of the runs (Figure 3), except for a significant period at the end of run 1. The results at the end of run 1 are unexpected since the north reactor panel continued to receive more sunlight than the south reactor panel and the dilution rate remained consistent with previous days. A practical error likely occurred before sampling and determination of the biomass concentrations. For instance, it was found that in some cases biofilm within the panels or tubes was released into the cultures as a result of cleaning or friction, which ultimately influenced the measured biomass concentrations and gave an incorrect representation of the sampled cultures. This issue was resolved in subsequent runs by cleaning the biofilm in the reactor panels hours before diluting and sampling.

In Table 5, an overview of the average biomass productivity in the individual reactor panels and the prototype is given for the four batch dilution runs. These average biomass productivities were determined for the pseudo-steady-state periods of each run, during which the daily reactor dilution was constant over several subsequent days (Figures 3A–D; Table 5).

For the north reactor panel, the average biomass productivity increased with decreasing daily reactor dilution and increasing biomass concentration, up to a dilution of 0.34 days^−1^ which was applied during run 3 (Table 5). In this run, the average biomass productivity was 0.43 g L^−1^ d^−1^ (Table 5). At a lower daily reactor dilution, which was applied during run 4, the biomass concentration increased (Figure 3D) and the average biomass productivity decreased (Table 5). In this run, the denser culture may have been more photo-limited due to self-shading of microalgal cells, resulting in larger dark zones and lower biomass productivities.

In the south reactor panel, the highest average biomass productivity of 0.31 g L d^−1^ was achieved in run 4, during which the lowest daily reactor dilution of 0.16 days^−1^ was applied and the highest biomass concentrations were measured (Table 5). A similar average biomass productivity was achieved in run 2, during which a higher daily dilution of 0.44 days^−1^ was applied and lower biomass concentrations were measured (Table 5). During this run, the lowest daily irradiance was measured on the south reactor panel (Figure 3F). In that case, the higher daily dilution and lower biomass concentration in the panel could have been favorable for biomass productivity. In run 1, during which the highest daily dilution of 0.53 days^−1^ was applied and the highest irradiance was measured on the south reactor panel, a lower biomass productivity was achieved. More wash-out of biomass likely occurred, and the thinner culture may have been more subjected to photo-inhibition.

Between the four runs, the differences in average biomass productivities were up to 0.18 g L^−1^ d^−1^ in the north reactor panel and up to 0.08 g L^−1^ d^−1^ in the south reactor panel (Table 5).

Furthermore, an average areal biomass productivity of 17–23 g m^−2^ d^−1^ was achieved in the prototype during the batch dilution runs (Table 5). The highest areal biomass productivity was found during run 4, during which the lowest daily reactor dilutions were applied in the panels (Table 5). In this run, the average photosynthetic efficiency was 3.1%. The biomass productivities and photosynthetic efficiencies obtained during the batch dilution runs were lower compared to results obtained during the continuous dilution runs (Table 3, 5; Figure 5).

### 3.3 Photosynthetic parameters

During each of the batch dilution runs, the maximum photosynthetic rate and the dark-adapted quantum yield of PSII photochemistry of the cultures in the reactor panels were determined at multiple times of the day and different temperatures.

#### 3.3.1 Maximum photosynthetic rate

The maximum specific oxygen production rates were generally higher in samples from the north reactor panel than in samples from the south reactor panel (Table 6). Possibly the algal culture in the north panel expressed a higher photosynthetic capacity because this culture was exposed to higher light intensities. The highest maximum specific oxygen rates were found during run 4 (Table 6), in which the lowest daily reactor dilution was applied and the highest biomass concentrations were measured (Table 5; Figures 3A–D). At these reactor dilutions and biomass concentrations, photoinhibition may have been limited due to the formation of dark zones in the denser cultures. In that case, individual microalgal cells spent more time in the dark compared to cells in lighter cultures, which may have allowed the microalgae to recover from the effects of high light intensities at the reactor surface. The maximum specific oxygen rate was found to decrease with increasing reactor dilution (Table 6). At higher daily reactor dilution, for instance in run 1, the biomass concentrations in the reactor panels were relatively low (Figure 3A) and microalgal cells were exposed to a sudden and more significant increase in exposure to sunlight after dilution, which may have resulted in photoinhibition and affected the photosynthetic capacity of cells.

**TABLE 6:** The average maximum specific oxygen rate, the initial slope of the photosynthesis-irradiance curve, and the dark-adapted quantum yields of PSII photochemistry of the cultures from the reactor panels during the batch dilution runs. Standard deviations are also given.

	Maximum specific oxygen production rate [μmol O_2_ g_x_^−1^ s^−1^]		Initial slope of PI curve [x 10^−3^]		Dark-adapted QY of PSII photochemistry [-]	
	North panel	South panel	North panel	South panel	North panel	South panel
Run 1	1.21 ± 0.74	1.72 ± 0.33	4.80 ± 2.53	6.70 ± 1.96	0.65 ± 0.01	0.66 ± 0.03
Run 2	2.42 ± 0.54	2.34 ± 0.63	5.97 ± 1.04	7.70 ± 1.58	0.67 ± 0.06	0.69 ± 0.04
Run 3	3.06 ± 0.63	2.39 ± 0.73	9.31 ± 1.24	10.6 ± 2.76	0.69 ± 0.02	0.71 ± 0.02
Run 4	3.41 ± 0.93	3.02 ± 1.15	12.5 ± 3.19	12.6 ± 3.78	0.69 ± 0.01	0.71 ± 0.01

**FIGURE 4:**
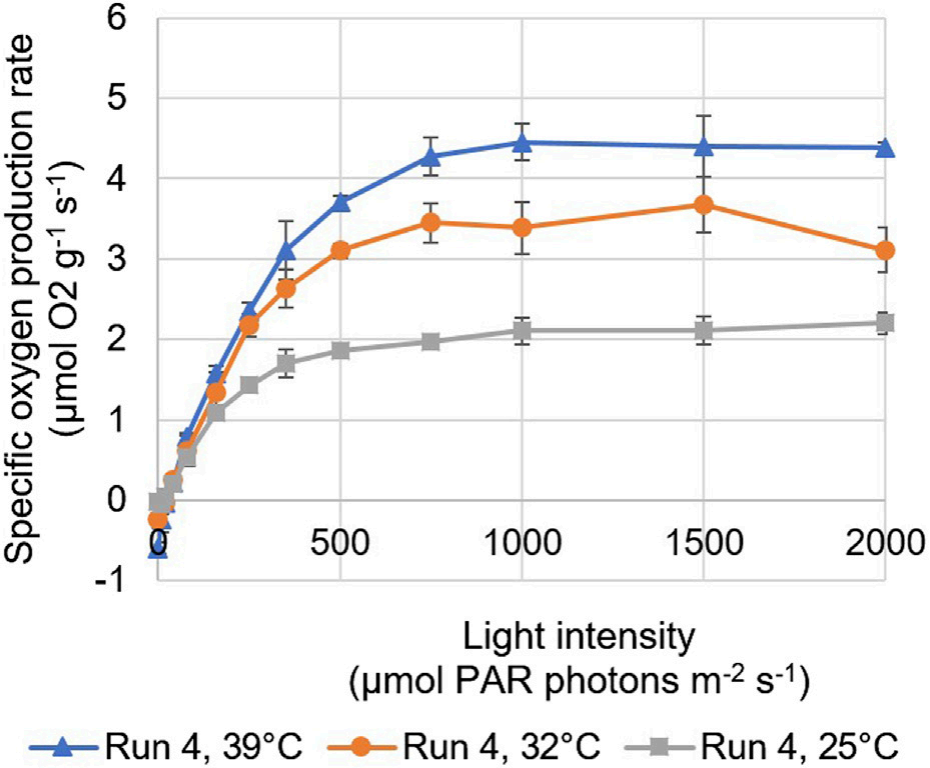
Photosynthesis-irradiance (PI) of samples taken from the north reactor panel during run 4, based on BOM measurements performed at temperatures of 25, 32, and 39°C. The error bars indicate the standard deviation of the measurements.

The maximum specific oxygen production rate was found to increase with temperature; the highest values were found for measurements performed at a temperature of 39°C, which is the optimal temperature of *Picochlorum* sp. (Figure 4). At temperatures below 39°C, reduced enzymatic reactions are reflected in lower rates of photosynthetic oxygen evolution (Figure 4). Moreover, no significant differences were found between the measurements of samples taken at different times of the same day, except during run 1. In this run, the highest daily reactor dilution was applied and the lowest biomass concentrations were measured (Figure 4). An increase in the maximum specific oxygen production rate was found in time, as the biomass concentration in the reactor panels increased during the day and the effects of photoinhibition in the culture likely decreased.

Similar correlations were found for the initial slopes of the PI curves (Table 6). These initial slopes can be seen as the product of the yield of oxygen (i.e., photosynthesis) on light and the specific light absorption coefficient. The slopes were found to increase with decreasing daily reactor dilution and increasing biomass concentrations (Table 6; Figures 3A–D). In runs where lower daily reactor dilutions were applied and higher biomass concentrations were measured (Figures 3A–D), microalgal cells may have acclimated to lower light conditions in the darker zones of the culture by increasing their pigmentation, and as such the specific absorption coefficient. Also, cells may have been less subjected to photoinhibition, resulting in a higher yield of oxygen on light. During most runs, the initial slope was larger for samples taken from the south reactor panel compared to samples taken from the north reactor panel (Table 6). This may have been caused by a higher degree of photoacclimation and reduced photoinhibition in the south reactor panel, as this panel was exposed to less sunlight. Moreover, the highest slopes corresponded to measurements performed at the optimal temperature (Figure 4). No significant differences were found between the initial slopes measured for samples taken at different times of the same day during almost all runs.

#### 3.3.2 Dark-adapted quantum yield of PSII photochemistry

The dark-adapted quantum yield of PSII photochemistry represents the maximum efficiency with which light energy is converted into chemical energy through photosynthesis, and is thereby used as a photosynthetic performance indicator.

During the batch dilution experiments, higher dark-adapted quantum yields of PSII photochemistry were found during runs in which lower dilution rates were applied and higher biomass concentrations were measured (Table 6). The highest dark-adapted quantum yield of PSII photochemistry that was obtained was 0.73 (Table 6), which is a typical value for healthy microalgal cells. Quantum yields were found to decrease with increasing dilution rates. In both reactor panels, the lowest quantum yields (0.65 in the north reactor panel and 0.66 in the south reactor panel) were found during run 1, in which the highest daily reactor dilution was applied (Table 6). In this run, microalgal cells were likely more subjected to photoinhibition, as a result of the lower biomass concentrations (Figure 3A) and the larger increase in light exposure after dilution.

Moreover, slightly higher quantum yields were found for cultures in the south reactor panel during all runs (Table 6), as this reactor panel received less sunlight and cultures were likely less subjected to photoinhibition compared to cultures in the north reactor panel. No significant differences were found between the measurements of samples taken at different times on the same day. Also, no significant differences were found between measurements performed at different temperatures, since measurements were based on physical, temperature-independent reactions.

## 4 Discussion

**FIGURE 5:**
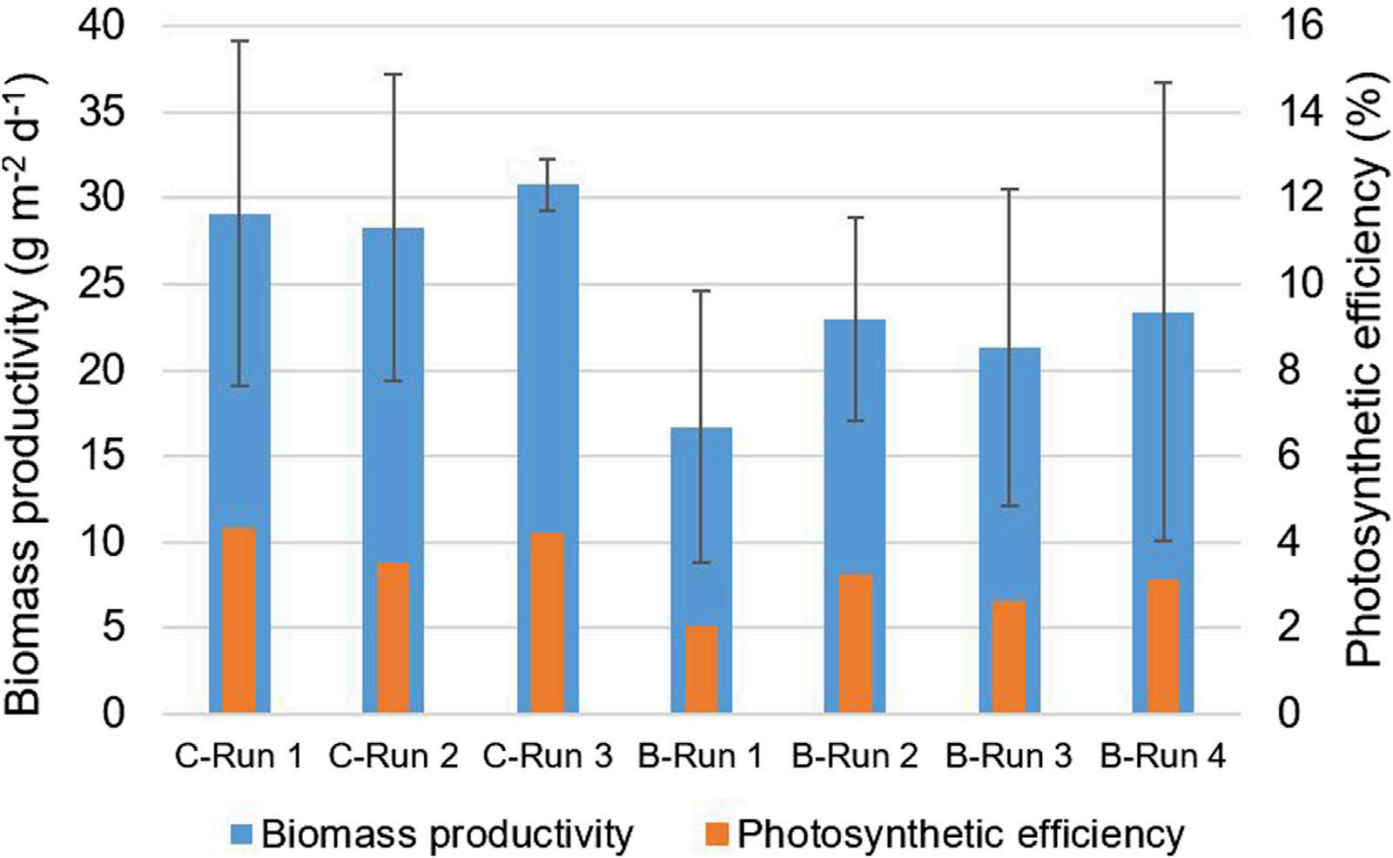
The average areal biomass productivity (g m^−2^ d^−1^) and the photosynthetic efficiency (%) observed during the continuous dilution (C) and the batch dilution (B) runs. The error bars indicate the standard deviation during a run.

In this study, the cultivation of microalgae in a novel V-shaped photobioreactor was investigated using a prototype that was operated under outdoor conditions on Bonaire (12°N, 68°W) for several months. The V-shaped photobioreactor design was proposed in Chin-On et al. (2022) but has never been studied in practice. The novel design aims to efficiently capture and dilute sunlight by covering the entire ground surface area and enabling light refraction at the inclined reactor surfaces. When more light at a lower intensity is available in a microalgae culture, higher biomass productivities and higher biomass yields on light can be achieved. In many other photobioreactor systems, sunlight is lost to the ground in between reactor units, and/or high light intensities around midday negatively affects the growth of microalgae as a result of photo-saturation and inhibition. While the V-shaped photobioreactor design can improve the areal biomass productivity, the overall costs (e.g., including material costs) still need to be analyzed. Nevertheless, the goal of our current study was to determine the biomass productivity in a V-shaped photobioreactor in practice and to analyze the growth of the microalgae under outdoor conditions. The experimental results of this study showed that the novel photobioreactor design of the prototype enabled sunlight at midday to be significantly diluted to lower light intensities (Tables 2, 4), which are more favorable for microalgal growth. In this manner, the reactor design aided in reducing the effects of photoinhibition and allowed for high yields of biomass on the available light. High biomass productivities and photosynthetic efficiencies of 28–31 g m^−2^ d^−1^ and 3.5%–4.3% were achieved during the continuous dilution experiments (Figure 5) using the native *Picochlorum* sp. *(BPE23)* microalgae strain. The cultivation of *Picochlorum* sp. was achieved without any form of temperature control, despite large temperature fluctuations in the reactor panels during the day and high reactor temperatures of 35°C–45°C at midday (Table 2). The results therefore also emphasize the robustness and thermotolerance of *Picochlorum* sp. The high biomass productivity and the absence of active temperature control, which is a necessity in most photobioreactor systems, are ultimately beneficial for reducing the costs of microalgae production. The obtained biomass productivities in the continuous dilution experiments (i.e., 28–31 g m^−2^ d^−1^) are relatively high compared to long-term results achieved in other studies on outdoor microalgae cultivation in photobioreactors. For instance, in De Vree et al. (2015), average biomass productivities of 19.4 and 20.5 g m^−2^ d^−1^ were reported for the cultivation of *Nannochloropsis* sp. in outdoor vertical tubular and flat panel reactors in the Netherlands, respectively. In those experiments, average photosynthetic efficiencies of 2.4% and 2.7% were achieved (De Vree et al., 2015). Another example is the study presented in (Paul et al., 2022), in which *Nannochloropsis* sp. was cultivated in seawater in an outdoor raceway pond in India for a year. The outdoor conditions were characterized by a broad temperature range (15°C–40°C) and fluctuating light conditions (Paul et al., 2022). Ultimately, an average biomass productivity of 20 g m^−2^ d^−1^ and an average photosynthetic efficiency of 2.0%–2.4% were achieved (Paul et al., 2022). In (Morillas-España et al., 2020), biomass productivities of 10–35 g m^−2^ d^−1^ were obtained during year-long cultivation of *Scenedesmus almeriensis* in a thin layer cascade photobioreactor in a greenhouse in Almeria, Spain.

Biomass productivities that were achieved in our study are also relatively high compared to those achieved in other novel outdoor photobioreactors. For instance, in Díaz et al. (2021), a Fibonacci-type photobioreactor was scaled up to cultivate *Dunaliella salina* in the Atacama Desert without the use of cooling systems. Biomass productivities of up to 2.41 g m^−2^ d^−1^ were measured (Díaz et al., 2021). Moreover, in Lee et al. (2022), an easy-to-scale-up photobioreactor made of polymer film was developed to cultivate cyanobacteria. Biomass productivities of up to 17.8 g m^−2^ d^−1^ were reached (Lee et al., 2022). While our results are promising, it must be noted that the biomass productivities obtained in different studies are not directly comparable to each other or the results of our study, as many factors can differ, such as light availability and intensity, temperature, microalgal strains, reactor dimensions, the time of the year, and other cultivation or operational conditions.

In this study, the biomass productivities obtained during the batch dilution experiments (i.e. 16.7–23.4 g m^−2^ d^−1^) were lower compared to the productivities achieved in the continuous dilution experiments (Figure 5), despite the slightly higher availability of sunlight, and higher temperatures during the batch dilution runs (Tables 2, 4). Biomass productivities were likely affected by the daily dilution procedure. During the batch dilution experiments, a fraction of the culture in each reactor panel was harvested each afternoon, after which the reactor panels were refilled with medium. Harvesting and refilling required more time the higher the daily reactor dilution; for the first two runs up to 1–1.5 h were needed. During this time, not all incident sunlight on the reactor panels was used for microalgae production. This loss of light was estimated to be 3%–6% of the total daily incident sunlight during the first and second runs.

Furthermore, during the batch dilution experiments, microalgal cells in the reactor cultures may have been exposed to light stress after reactor dilution. After reactor dilution each day, the biomass concentration of the cultures in the reactor panels dropped and individual cells in the cultures were suddenly exposed to higher light intensities. This change may have inactivated or damaged the photosystems of the microalgae and affected photosynthesis as a result of photoinhibition. In addition, during the first batch dilution run, a sudden decrease in culture temperatures of up to 10°C was also observed after dilution since the reactor panels were refilled with refrigerated medium. This drop in temperature may have slowed the growth of the microalgae (Ras et al., 2013). In the continuous dilution experiments, sudden light and temperature shocks were avoided as the dilution of the cultures was distributed over the daytime hours.

From the measurement of several photosynthetic parameters, light and temperature were indeed found to influence the photosynthesis of the microalgae. The maximum specific oxygen production rate, the initial slope of PI curves, and the dark-adapted QY of PSII photochemistry were found to increase among the batch dilution runs (Table 6), as the daily reactor dilutions decreased and the biomass concentrations in the reactor panels increased. At lower daily reactor dilutions and higher biomass concentrations, light shocks after dilution were smaller, cultures were likely less subjected to photoinhibition due to the formation of darker zones, and cells may have increased their pigmentation to acclimate to lower light conditions. In addition, the maximum specific oxygen production rate and the initial slope of PI curves were found to increase with temperature up to the optimal temperature of 39°C for *Picochlorum* sp. Higher temperatures could not be tested with the BOM.

The measured maximum specific oxygen production rates of the cultures (Table 6), especially those measured during run 4 of the batch dilution experiments, were relatively high compared to data presented in literature for the same *Picochlorum* strain. In a previous study (Barten et al., 2022), the maximum specific oxygen production rates at 39°C were found to be below 3 μmol O_2_ g^−1^ s^−1^ for *Picochlorum* sp. *BPE23* grown on urea as well as on nitrate, from which maximum specific growth rates of 4.98 days^−1^ and 3.79 days^−1^ were estimated, respectively. In run 4 of our study, the maximum specific oxygen production rates were found to be above 4 μmol O_2_ g^−1^ s^−1^ for both cultures when measured at 39°C (Figure 4; Table 6). In this run, the lowest daily reactor dilutions were applied, and the highest biomass concentrations were measured in the panels. The differences between our results and the previously reported values in (Barten et al., 2022). are unexpected, as our cultures were cultivated under outdoor conditions, which were more variable and less optimal in terms of light and temperature compared to conditions in the laboratory experiments ((Barten et al., 2022). Microalgal cells in our reactor cultures may have adapted in time to the conditions to which they were exposed, which could have resulted in higher photosynthetic rates.

The maximum specific oxygen production rates that were obtained for *Picochlorum* sp. in this study are also high compared to rates reported for other microalgae, such as *Nannochloropsis* sp. and *Neochloris Oleoabundans* (Barten et al., 2022). Other species of *Picochlorum* have been characterized by their high growth rates as well, in addition to their ability to grow well under broad and changing conditions (Weissman et al., 2018; Krishnan et al., 2021). In line with such observations, our results highlight the potential of *Picochlorum* sp. for fast growth and high biomass productivities under outdoor conditions at a low-latitude location. Nevertheless, the effects of extreme temperatures and photoinhibition must be further investigated and taken into account. A V-shaped photobioreactor can reduce the occurrence of such effects by diluting sunlight at the reactor surface.

## Data Availability

The raw data supporting the conclusion of this article will be made available by the authors, without undue reservation.

## Supplementary material

SUPPLEMENTARY FIGURE S1 Technical drawing and dimensions of the prototype.

SUPPLEMENTARY FIGURE S2 Sequence of light intensities and time intervals for BOM measurements.

SUPPLEMENTARY FIGURE S3 An example of measured irradiance and temperature during the day.
