# Supplementary material for: Outdoor cultivation of Picochlorum sp. in a novel V-shaped photobioreactor on the Caribbean island Bonaire
Source: Front Bioeng Biotechnol. 2024 Jun 13;12:1347291. doi: 10.3389/fbioe.2024.1347291 (PMC11208710; doi:10.3389/fbioe.2024.1347291)

# Supplementary Material 3. An example of measured irradiance and temperature during the day

In the figures below, the irradiance and temperature measured on day 4 of run 1 of the continuous dilution experiments are shown as an example to illustrate the variation during the day.


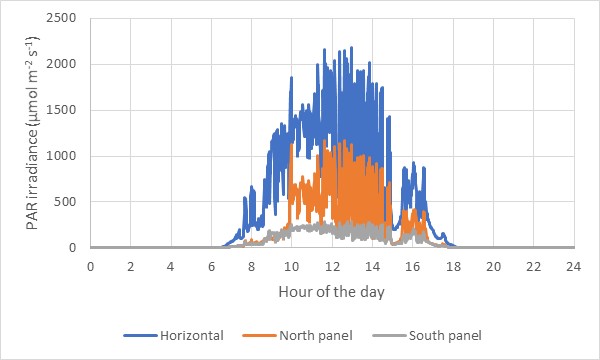


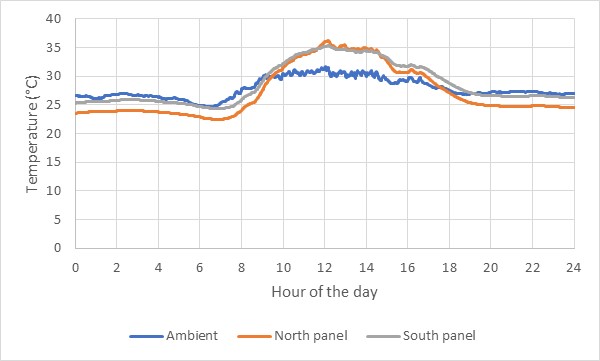

Supplement: Supplementary file 1 [file DataSheet3.DOCX]
