# Supplementary material for: Outdoor cultivation of Picochlorum sp. in a novel V-shaped photobioreactor on the Caribbean island Bonaire
Source: Front Bioeng Biotechnol. 2024 Jun 13;12:1347291. doi: 10.3389/fbioe.2024.1347291 (PMC11208710; doi:10.3389/fbioe.2024.1347291)

# **Supplementary Material 2. Sequence of light intensities and time intervals for BOM measurements**


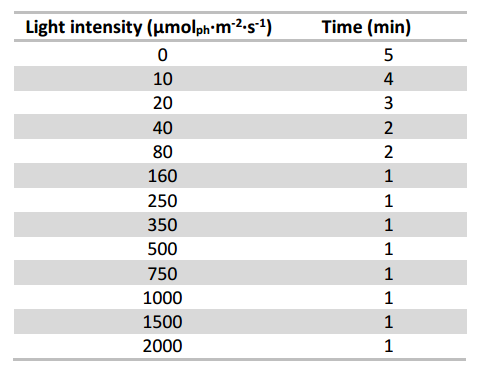

Supplement: Supplementary file 2 [file DataSheet2.DOCX]
