# Supplementary material for: Outdoor cultivation of Picochlorum sp. in a novel V-shaped photobioreactor on the Caribbean island Bonaire
Source: Front Bioeng Biotechnol. 2024 Jun 13;12:1347291. doi: 10.3389/fbioe.2024.1347291 (PMC11208710; doi:10.3389/fbioe.2024.1347291)
Supplement: Supplementary file 3 [file DataSheet1.DOCX]

# **Supplementary Material 1. Technical drawing and dimensions of prototype**


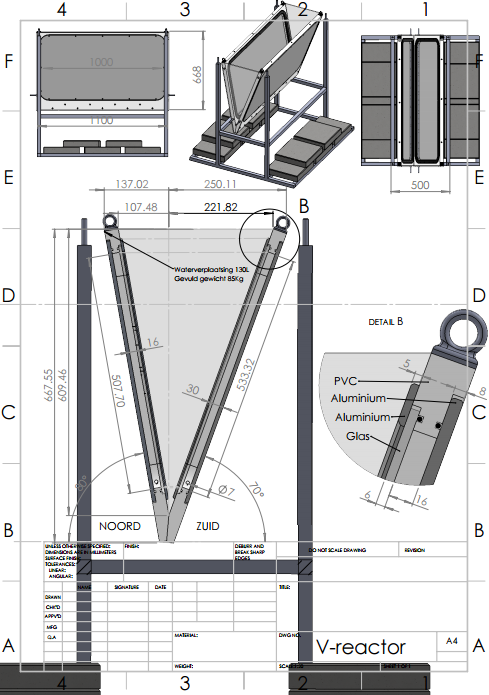


Supplementary Figure 1. Technical drawing of the V-shaped photobioreactor prototype

Supplementary Table 1. Dimensions of the V-shaped prototype and the individual reactor panels. ‘North reactor panel’ refers to the reactor panel pointing in the north direction, and ‘South reactor panel’ refers to the reactor panel pointing in the south direction.

|  | V-shaped prototype | North reactor panel | South reactor panel |
| --- | --- | --- | --- |
| Area – horizontal [m^2]^ | 0.33 | 0.11 | 0.22 |
| Length – horizontal [m] | 1.0 | 1.0 | 1.0 |
| Width – horizontal [m] | 0.33 | 0.11 | 0.22 |
| Height – vertical [m] | 0.5 | 0.5 | 0.5 |
| Height – diagonal [m] | - | 0.51 | 0.53 |
| Volume – total [L] | 21 | 10 | 11 |
| Volume – panels [L] | 16 | 7.6 | 8.3 |
| Volume – recirculation [L] | 5.6 | 2.8 | 2.8 |
